# Editorial and Miscellaneous

**Published:** 1855-09

**Authors:** 


### EDITORIAL AND MISCELLANEOUS.

### SALUTATORY.

The projectors of the "*Atlanta Medical and Surgical Journal*" have not undertaken the enterprise without counting the cost, both of labor and money, and are fully prepared and determined to encounter the difficulties that may be presented, relying upon the friends of medical advancement in this whole region of country to sustain them, not only or simply by pecuniary aid in the way of subscriptions, but still more decidedly by contributions from their pens.

Our object is to offer to the profession an open and free medical press—to offer to our brethren an accessible medium through which they can communicate with the public, and especially to aid in building up a domestic, and particularly, a *Southern Medical Literature.*

There is no expectation of deriving pecuniary profit from the publication, but only to make it self-sustaining. To do even this, will require a considerable subscription list, but from the already decided manifestations of interest which we have had from numerous quarters, in our whole series of operations, *to establish in the city of Atlanta a seat of medical learning*, we do not doubt that a sufficient patronage will be extended to the effort to make it certain that it will not be discontinued.

The object of this Journal will be to advance the claims of *true medicine*, rather by the presentation and diffusion of truth, than by assailing error, except incidentally. We shall not, therefore, set ourselves up as infallible judges of merit, or as censors of the communications with which our friends may be disposed to furnish us ; at the same time, we wish it distinctly understood that we do not hold ourselves responsible for the views which any correspondent may entertain ; and while we anxiously desire to have among our contributors a long list of scientific men, who have already established for themselves a reputation, we hope to be the instrument of aiding in building up the just fame of many who have not yet entered the lists; and we now offer our Journal as the organ of communication for all true medical men, who desire to make it so, and if among our contributors we should stumble upon some who are not as wise as some others may consider themselves to be, they shall still have courtesy and kindness at *our* hands.

The course of this Journal has already been indicated by the example of its projectors, in the quiet, even, but firm and fixed purpose manifested in their whole line of advance towards the accomplishment of the objects in view, turning neither to the right or to the left, having neither time or taste for controversy, but being willing to be judged by their deeds, and to submit to the verdict of a just and enlightened public.

We, as their representatives, in the conduct of this Journal, have only to point to their example and success as an indication of what may happen, from what has happened; and this, with "Peace and Science, but Truth without Fear" as our motto, we announce as fully "defining our position."

---

### ATLANTA MEDICAL COLLEGE.

We propose in this notice to give, in as few words as possible, the history, prospects, &c., of the Atlanta Medical College.

Before commencing this synopsis, we will insert the charter of the above institution, which, as will be seen below, was approved by the Legislature of Georgia on the 14th of February, 1854:

*An Act to incorporate the Atlanta Medical College, and for other purposes therein mentioned.*

Section I. *Be it enacted* by the Senate and House of Representatives of the State of Georgia in General Assembly met, and it is hereby enacted by the authority of the same, that L. C. Simpson, Jared J. Whitaker, John Collier, Hubbard Cozart, Daniel Hook, John L. Harris, William Herring, Green B. Haygood, and James M. Calhoun, trustees, and their successors in office, be and the same are hereby constituted a body politic and corporate, under the name and style of the "Trustees of the Atlanta Medical College," that they may have a common seal, a perpetual succession of officers and members, and are hereby declared capable of suing and being sued, pleading and being impleaded, answering and being answered unto; and said corporation may take, have, possess, and acquire by gift, grant, purchase, bequest or devise lands, tenements, hereditaments, goods, chattels, and other estates, acquired by said corporation, it [to?] have, use, improve, and convey, and to keep a book of records for registering all the diplomas granted by them under the provisions hereinafter mentioned.

Sec. II. *Be it further enacted* by the authority aforesaid, That the said corporate body are hereby authorized and empowered to elect all such officers as may be necessary, and pass all such by-laws, rules and regulations as may be necessary to carry into effect the objects of their associations : *Provided*, That such by-laws, rules, and regulations be not contrary to the Constitution and laws of this State and the constitution of the United States ; and in case of death, removal, resignation, or refusal to act, of any member of said Board of Trustees, the said body corporate or a majority of them shall have power to fill such vacancies.

SEC. III. *Be it further enacted* by the authority aforesaid, That the said Board of Trustees are hereby authorized to establish a Medical College in the city of Atlanta, on such principles, and under such rules and regulations, and with such Professors as may in their judgment be best calculated to perpetuate the same, and promote the improvement of its pupils in the several branches of the science of medicine.

SEC. IV. *Be it further enacted* by the authority aforesaid, That a majority of the members of the Board of Trustees shall constitute a quorum for the transaction of business, but that a less number may adjourn from day to day until a quorum shall attend.

SEC. V. *Be it further enacted*, That any three or more of said Trustees above named are hereby authorized to call [a ?] meeting of the Board at any time they may think proper, to be held in the City of Atlanta, for the purpose of making and establishing such by-laws as are necessary, and for the purpose of transacting any other business that may be necessary to be done.

SEC. VI. *Be it further enacted* by the authority aforesaid, That the Trustees, together with the regular Professors, shall constitute a Board, who are hereby authorized and empowered to confer the Degree of Doctor of Medicine upon such applicants, in such manner, and at such times, and under such circumstances, as may to the said Board seem fit and proper: *Provided*, The applicants shall have attended two full courses of lectures in said College, or one in said College and one in some other respectable Medical College or University.

SEC. VII. *And be it further enacted* by the authority aforesaid, That said Board of Trustees shall have power to fix the times for commencement and closing of the lectures each year.

SEC. VIII. *And be it further enacted* by the authority aforesaid, That all laws and parts of laws militating against this act, be and the same are hereby repealed.

Approved, February 14th, 1854.

Soon after the approval of the above charter, the Board of Trustees had their first meeting, and at this, or some subsequent meeting, set apart the 15th day of June, of the same year, to select Professors to fill the various chairs.

The plan proposed and adopted to have the chairs filled may be best shown by the following extract from an advertisement extensively circulated prior to the election : "The Trustees of said institution meet on the 15th day of June next for the election of Professors for said College, and to secure the best talents of the country, do invite applications for the same from the eminent men of the profession generally ; at which time applications with the testimonials of character and qualificarions will be impartially considered, and the election made according to the best judgment of the Trustees."

On the 15th of June, the day alluded to in the above extract, the Board of Trustees met, and after duly considering the numerous applications, selected Professors to the several chairs.

As will be seen by the charter above, it is left entirely optionary with the Trustees and Faculty whether they adopt winter or summer sessions.

At the first joint meeting of Trustees and Faculty this feature of the enterprise was considered, and unanimously decided in favor of summer sessions.

The first annual announcement of this institution was issued in January of the present year.

From the complete organization, in June, 1854, up to the commencement of the present course of lectures, several changes have occurred in the Faculty from death, resignation, &c., all of which have been duly announced.

The Introductory Address to the present and first course of Lectures in this institution was delivered on the first Monday in May last by JOHN W. JONES, M. D., Professor of the Theory and Practice of Medicine. From this very able and appropriate address we must be permitted to make at least one extract, as it gives fully our position, and is, we feel confident, an expression of the feelings of every member of the Faculty. In speaking of our competitors, prospects, &c., he says:

"To the formidable competitors by which our institution is in close proximity surrounded, we are ready to extend the right hand of fellowship, and if the greeting be reciprocal, to salute them as honored co-laborers in a common cause, and whose noble achievements will only excite in us the spirit of professional emulation—cheerfully yielding all that is due to seniority, and only asking in return 'honor to whom honor is due;' and promising on our part faithfully to 'render unto Cæsar the things that are Cæsar's.' And while on this subject, we would further remark, that we fully coincide with the sentiments of a distinguished teacher of the oldest, and certainly one of the most reputable, Medical Colleges in America—we allude to Professor Wood, of the University of Pennsylvania. He says: 'A race is before us—a noble prize is to be won; we hail every honorable competitor with a friendly spirit. The very excitement of a fair and open contest is equivalent almost to victory. Let each school present its advantages in the strongest light, and exert its own strength to the utmost, leaving to its neighbor the same privilege, unmolested, and whichever may maintain precedence in the struggle, no just or honorable spirit will complain.' This is the language of one of the distant successors of the venerable Rush, and it erects a platform of College ethics on which we willingly take our stand; and we will only add, that had this Faculty no other incentives to indorse and conform to, this honorable and equitable standard of neighborship, that the sanctity of its position and the source whence it derives its existence and authority, would be amply sufficient to constrain it in all things whatsoever to 'act worthy of the high vocation wherewith it has been called.' Thus actuated, as we trust by motives of philanthropy more than by the expectation of pecuniary aggrandizement, as well as by an ardent devotion to our time-honored profession, that neither acknowledges or feels any abatement, and by an invitation from the corporators of this Institution, we have assembled in your young but flourishing and rapidly growing city, which has justly become the pride of Georgia, and the wonder and admiration of her sister States. We have come up to this great centre of our sunny South to take our respective stations in a temple, if not dedicated, for the present appropriated, to the great and good cause of THERAPIA—and here, from day to day, to dispense to the self-sacrificing and uprising sons of our country further light in the 'healing art;' and here, from hour to hour, upon the sacred altar of HYGEIA, to lay our free-will offerings, and to minister in a cause only second to Christianity itself. And now, after numerous hindrances and disappointments, which have materially retarded the progress of our preparation, the hour of our embarkation has at last arrived—our vessel (to use a figurative expression) has been pronounced reliable and seaworthy by competent inspectors—our crew and cargo all on board, and our banner unfurled to the breeze, we dispense with our moorings and steer directly into the deep and broad ocean of Medical enterprise!

Whether we are destined to be wafted by auspicious breezes upon the smooth waters of prosperity and success, or to be driven before the unrelenting fury of adverse winds, amidst billows and breakers, time alone will determine. It may be that we shall have to navigate the turbid quick-sadds of Envy and Vituperation, and to cruise along the precarious reefs of Disparagement and Detraction. Be it so: 'the die is cast,' and no matter if a Scylla appears on our starboard and a Charybdis on our larboard, still we will not vary our bearings or change our course. Our watchword is Onward! and onward we will move, ever bearing in mind that it is

"'In the rough school of billows, storms and clouds,
Nursed and matured, the pilot learns his art;
Thus fates dread ire by many a conflict forms
The lofty spirit and enduring heart."

And the minor obstacles to which we have alluded, and which, if we have to meet, will but serve to test our strength and to redouble the energy of those on board. Be this as it may, and happen what will, honored as we are with helmsmen's places on this our trial voyage, whether we sink or swim the cause of our vessel shall be our cause—her fate shall be our fate. And remember, too, that we hail from the central harbor of the South, with a commission and passport, plenipotentiary in all respects, bearing upon their face the honored signet and broad ægis of 'Wisdom, Justice, and Moderation." Thus authorized and thus equipped we sail from the known to the unknown, and enter upon the expedition not without apprehensions, and yet full of hope; and whether destined to perish from calm or storm, our last wish will be that enough of our gallant mates may suruive the wreck qualified and willing to succeed us in command, and our last words will be those of the intrepid and dying Lawrence, 'Don't give up the ship!'"

And now, when we have it in our power to announce a safe return from this "trial" and prosperous voyage, in no boasting spirit, but with confidence and gratitude, we hesitate not to enter fully the field of a world-wide and honorable competition, determined that no effort of ours shall be wanting to sustain and *advance* the successful position already attained.

It will not, however, fail to be a regular part of our duty to inspect the vessel before renewing the voyage, and to furnish it with all the necessary or desirable means and appliances which science and art are so rapidly and profusely furnishing; and with an energy and determination only *increased* by successful experiment, we announce ourselves as *candidates for the front rank* in medical enterprize.

Our institution was opened under the most unfavorable circumstances; at a time when there was a great pressure in the money market—such a pressure as we have not seen in Georgia since 1838. The distribution of our announcements, from various hindrances, was delayed until within three months and a half of the opening of the present session—by no means sufficient time to have it generally known that our school would certainly open the present summer. Various reports were put in circulation, derogatory to our interests, by those who, from disappointed hopes, &c., felt an antipathy to the institution—reports that it was impossible to counteract before the commencement of the course.

So true is the above, that more than one student, now in attendance, came to Atlanta to learn whether there was really a course of lectures going on, and after learning that such was the case, went home, made such arrangements as were necessary, and returned. With all these difficulties, we now have seventy-eight regularly matriculated students, and had we received those who have applied for admission since the last of June, the time of closing our books, we would have added seven or eight others to the list. This number, considering the unfavorable circumstances above alluded to, is truly encouraging. There is only one other institution, we believe, in America that has commenced with a larger class than has the Atlanta Medical College.

It is not, however, the number alone of which we feel so proud, and which so much encourages us, but the character, intelligence, and deportment of the young gentlemen composing the class. Had we only half the number we now have, and they, of such gentlemen as compose the present class, we would feel fully compensated for the many sacrifices we have made in building up this institution.

Judging from the past, what can we expect in the future but the most brilliant success? We are no prophets, but will here predict that our second class will double in number our first.

As regards location, we have, above all others, the point for a summer school. Atlanta is peculiarly adapted to summer sessions; the four months proposed for our course, viz: May, June, July, and August, are as healthy, if not the healthiest, four months in the year. Situated in one of the healthiest sections of our State, upon the high lands dividing the waters of the Gulf and those of the Atlantic, it is impossible for Atlanta ever to be visited by those formidable epidemics which occasionally so rapidly depopulate our sister cities. Surrounded by all that is conducive to health, the student may feel as secure from disease at this point as he would upon the loftiest peak of the Lookout. It is desirable, not alone in this particular, but, located at the termini of four principal railroads, which connect it directly or indirectly with almost every State in the Union, its accessibility is unsurpassed. As a central, acessible, and healthy point, it is beyond all doubt the first city in the Southern States.

The Corner Stone of our College Edifice was laid on the 21st of July last, upon which occasion we had a most appropriate and eloquent address from our talented young townsman, H. D. Beman, Esq.

The college building is now in progress of erection, and will be in readiness for the next course of lectures.

---

### FLEMING'S HYGIENIC JOURNAL.

This is the title of a monthly journal, the first number of which has been laid on our table. It is to be published in the city of Atlanta by Newton R. Fleming, M. D., at the very low price of two dollars a year. The objects contemplated in this enterprise, all must admit, should command for it a world-wide attention.

We cannot well conceive a subject of more importance than that embraced in the investigation of the laws which the Creator designed for the regulation of human health; and what is worthy of especial notice, in connection with this subject, is the fact that, in a large number of derangements of the human system which are but little amenable to the curative power of *medical treatment*, we have fully established the efficiency of *preventive measures.*

It cannot be questioned that it is the duty of medical men not only to be prepared to treat disease successfully when submitted to their hands, but also to be able and willing to enlighten the public as to the best course to be pursued to *keep out* of their hands; and addressing medical men principally, as we do, we would bespeak their support for this enterprise.

We agree fully in the idea suggested in the first number of this journal in reference to making the world fully acquainted. with all that is known of the nature and character of those agents, by which he is surrounded, bearing any relation to his physical or mental well being.

It is our doctrine that too much concealment and mystery has ever surrounded the science of Medicine and its collateral branches, and we are for exhibiting the whole truth, so far as a regard for the laws of propriety, having reference to our social relations, will allow.

As medical men we have nothing to lose, but everything to gain, by a universal diffusion of a knowledge of the *laws* which regulate the human system in health and disease, and we are perfectly satisfied that in proportion to the correct understanding of these principles, upon the part of the world, so will be the elevation of our *science* above the low arts of the charlatan and grossly criminal blunders of those who profess to relieve disease without the knowledge to fit them for the position.

The editor is evidently a man of talent and energy, and we wish him the fullest success in his philanthropic undertaking.

---

*Bunion.*—We have seen at Messrs. Metcalf & Co.'s a new application for the treatment of bunion. It is a very fine felt, like that used by piano-forte makers, about one-fourth of an inch in thickness, and covered on one side with an adhesive layer. A hole is punched through the felt, corresponding to the tumor. The lower surface of the felt being moistened, is applied to the skin, to which it adheres, while the bunion is protected from pressure by the thickness of the material. There is nothing new in the principle of this mode of treatment; it is only the nicety and convenience of the material to which we wish to call attention.—*Boston Med. and Surg. Jour.*

### ALEXANDER MEANS, M. D.

It is with pleasure that we have it in our power to announce the accession of Professor ALEXANDER MEANS to the chair of Chemistry and Pharmacy in the Atlanta Medical College. We presume that it is a generally admitted fact that for profound and thorough knowledge of the subjects embraced in his chair, and for eloquence as a lecturer, he has no superior in the United States. He will enter upon the discharge of his duties at the commencement of the next course.

It is perhaps proper for us to say that the acceptance of a professorship in this institution will not sever his connection with the Medical College of Georgia, at Augusta, but that he still retains his position in that deservedly prominent institution.

---

### BOOK NOTICE.

*The Pocket Formulary and Physicians' Manual.* By THOS. S. POWELL, M. D., Sparta, Georgia.

This is the title of a small work, a copy of which has been laid upon our table, and we can truly say of it "*multum in parvo.*"

It embraces the art of combining and prescribing medicines, with many valuable formula, tables, &c., which would doubtless be exceedingly useful to the physician in his daily practice.

It is intended to be constantly worn in the pocket of the practitioner, and contains a large amount of miscellaneous medical intelligence, especially useful to the young practitioner; also a number of valuable statistics, with the code of ethics adopted by the American Medical Association. We are assured that it has been highly approved by a number of prominent medical men in Philadelphia and elsewhere to whom it has been submitted, and we cordially give our endorsement to it, as a valuable addition to the medical literature of the country.

Its author is a gentleman of large and successful experience in the profession, and one to whom we shall look as a regular contributor to our Journal. It can be obtained from SMITH & EZZARD, of this city.

*Chloroform.*—In the *Annali Universali*, for May 1854, Dr. Turchetti calls attention to a great variety of cases, in which he has found local applications of chloroform beneficial. He says that whitlow can be arrested in six hours, by covering the affected part with compresses moistened by the anæsthetic liquid, the compresses being renewed every fifteen minutes. Mingled with belladonna ointment and applied to inflamed hæmorrhoidal tumors, chloroform is of incontestable utility; applied to the hypogastrium, or introduced into the vagina, it is serviceable in spasmodic dysmenorrhœa. In this case, it is sprinkled on layers of cotton. It is applied in the same way to cancerous tumors of the uterus and mamma, and not only alleviates pain, but prevents hæmorrhages. Slight sprain, spasmodic ischuria, and sympathetic bubo are the other affections in which Dr. Turchetti has observed the efficacy of chloroform applied topically.

---

*Cholera.*—M. Charcellay, Professor in the preparatory school of medicine at Tours, has addressed a memoir to the Imperial Academy of Medicine upon the subject of transmissibility of cholera in many localities of the district of Indre et Loire during the epidemics of 1832, 1849 and 1854.

---

*Tic Doloureux.*—Dr. Chisholm speaks in the highest terms of the benefits to be derived from the use of the ointment of veratria in neuralgia. He directs that it should be used in the proportion of fifteen or twenty grains to the ounce, and rubbed in until tingling and a peculiar pricking sensation is felt.

---

*Rheumatism.*—In the *Jour. de Med.* of Brussels, Dr. Hœr reports a number of cases of rheumatism, unaccompanied with much fever, but characterized by persistence in the swelling of joints and extreme pain, in which the tincture of *canabis indica*, in doses of eight to ten drops, *ter die*, removed the pain in a short time, this result being preceded by abundant diuresis.

